# Regional Impact of Climate on Japanese Encephalitis in Areas Located near the Three Gorges Dam

**DOI:** 10.1371/journal.pone.0084326

**Published:** 2014-01-03

**Authors:** Yuntao Bai, Zhiguang Xu, Jing Zhang, Deqiang Mao, Chao Luo, Yuanyuan He, Guodong Liang, Bo Lu, Michael S. Bisesi, Qinghua Sun, Xinyi Xu, Weizhong Yang, Qiyong Liu

**Affiliations:** 1 Division of Environmental Health Sciences, College of Public Health, The Ohio State University, Columbus, Ohio, United States of America; 2 Department of Statistics, The Ohio State University, Columbus, Ohio, United States of America; 3 Key Laboratory of Surveillance and Early-Warning on Infectious Disease, Chinese Center for Disease Control and Prevention, Beijing, China; 4 Chongqing Center for Disease Control and Prevention, Chongqing, China; 5 Wanzhou District Control of Diseases Prevention and Control, Chongqing, China; 6 Yichang Center for Disease Control and Prevention, Yichang, Hubei Province, China; 7 State Key Laboratory for Infectious Diseases Prevention and Control, National Institute for Viral Disease Control and Prevention, Chinese Center for Disease Control and Prevention, Beijing, China; 8 Division of Biostatistics, College of Public Health, The Ohio State University, Columbus, Ohio, United States of America; 9 Department of Vector Biology and Control, State Key Laboratory for Infectious Diseases Prevention and Control, National Institute for Communicable Disease Control and Prevention, Chinese Center for Disease Control and Prevention, Beijing, China; The Australian National University, Australia

## Abstract

**Background:**

In this study, we aim to identify key climatic factors that are associated with the transmission of Japanese encephalitis virus in areas located near the Three Gorges Dam, between 1997 and 2008.

**Methods:**

We identified three geographical regions of Chongqing, based on their distance from the Three Gorges Dam. Collectively, the three regions consisted of 12 districts from which study information was collected. Zero-Inflated Poisson Regression models were run to identify key climatic factors of the transmission of Japanese encephalitis virus for both the whole study area and for each individual region; linear regression models were conducted to examine the fluctuation of climatic variables over time during the construction of the Three Gorges Dam.

**Results:**

Between 1997 and 2008, the incidence of Japanese encephalitis decreased throughout the entire city of Chongqing, with noticeable variations taking place in 2000, 2001 and 2006. The eastern region, which is closest to the Three Gorges Dam, suffered the highest incidence of Japanese encephalitis, while the western region experienced the lowest incidence. Linear regression models revealed that there were seasonal fluctuations of climatic variables during this period. Zero-Inflated Poisson Regression models indicated a significant positive association between temperature (with a lag of 1 and 3 months) and Japanese encephalitis incidence, and a significant negative association between rainfall (with a lag of 0 and 4 months) and Japanese encephalitis incidence.

**Conclusion:**

The spatial and temporal trends of Japanese encephalitis incidence that occurred in the City of Chongqing were associated with temperature and rainfall. Seasonal fluctuations of climatic variables during this period were also observed. Additional studies that focus on long-term data collection are needed to validate the findings of this study and to further explore the effects of the Three Gorges Dam on Japanese encephalitis and other related diseases.

## Introduction

Japanese encephalitis (JE), which is caused by the Japanese encephalitis virus (JEV), is a mosquito-borne infectious disease that is prevalent throughout Southeast Asia and the Asian Pacific Rim [1], [2]. Approximately 67,900 cases of JE are reported annually, with an overall incidence rate of 1.8/100,000 per year worldwide; and approximately 33,900 (50%) of these cases occur in China [3]. Although the administration of vaccines in China has helped reduce its prevalence since 1980s, the disease continues to be a challenge for animal and human health in many areas of the country [4]–[7].

The incidence of JE has been reported annually in China since the establishment of a case reporting system in 1951. Since that time, two major JE epidemics have occurred: first, in 1966 when the annual incidence of JE rose to greater than 15/100,000 nationwide; and second, in 1971 when it rose again to 20.9/100,000 nationwide [8]. Beginning in the 1980s, the JE vaccination began dramatically decreasing both the number of cases of JE as well as its incidence rate [7], [9]. And, more recently, from 2000 to 2010, the reported number of cases decreased from 11,779 to 2,541 while the annual incidence rate decreased from 0.9/100,000 to 0.2/100,000 nationwide [10].

Nonetheless, JE continues to be a risk for human health. With the exception of the Xinjiang, Qinghai, and Tibet provinces, human JE cases have been reported throughout all of China. There are several reasons for this: first, residents have a low level of awareness of infectious diseases and therefore fail to get vaccinated [4], [11]; second, the virus has the capacity to mutate and therefore increase its resistance to available vaccines [12],[13]; third, the changes of human social behavior [14]. Most importantly, medicine to effectively cure the viral infection are still not available [15].

The emergence of JE involves a complicated interplay of hosts, vectors, climatic variables and anthropogenic factors [16], [17]. Human, cattle and horses are dead-end hosts, which could not replicate the virus to high enough titer to serve as a source for re-infection of mosquitoes [18]. All age groups could be infected by the JEV, but the elderly and children who are under 15 years of age are more susceptible to infection [5], [19]. JEV is transmitted to vertebrates by mosquitoes. The main vector of JE is *Culex. tritaeniorhynchus* in Asia, while the main vector is *Culex. annulirostris* in Australia (in the South Pacific area) [20]–[22]. In Asia, domestic pig acts as an amplifying host and holds a very important role in epidemiology of the disease in endemic areas [23], [24]. Migratory birds and bats may also play a role in spreading and reintroducing JEV [25], [26]. Birds in particular serve as the natural host of JEV, which therefore leads many to believe that JEV can never be completely eliminated due to their migratory nature [27]. Although JEV has been identified in over 90 wild and domestic bird species, ardeid wading birds are considered the primary enzootic hosts of JEV in some areas [26], [28], [29]. Several studies have shown that climatic variables like temperature, relative humidity, and rainfall are closely associated with the incidence of JE [30]–[32]. These climatic variables influence the transmission of JEV primarily through their effects on the virus cycle and vectors’ maturation in the stages of larvae and pupae [33], [34]. Social factors also play an important role in human JE epidemiology. A previous study showed that the major epidemic areas of JE in China moved from the eastern areas in the 1970s to the southwestern provinces in 2010. This is probably the results of the rapid economic development in the eastern areas [10]. Other potential factors that could influence the JEV transmission include elevation and the agricultural use of land [35], [36]. For example, rice agriculture is directly associated with the incidence of because rice paddies not only provide breeding sites for the vector, but also provide foraging sites for water birds that are susceptible to JEV infection [32].

Chongqing is located on the upper section of the Yangtze and is one of five provinces in China with a mean JE incidence rate higher than 1/100,000. Chongqing, along with Sichuan, Henan, Yunnan, and Guizhou has accounted for more than 50% of the total JE cases, in spite of the fact that they comprise only 26% of the total population of China [10].

The Three Gorges Dam (TGD) is a hydroelectric dam that spans the Yangtze River at Sandouping Island built in 2012. The construction of TGD has resulted in substantial changes to the depth and the flow pattern of the Yangtze River and has also led to the substantial development of farmland alongside the river path [37]. The negative impacts on the aquatic ecology of the Three Gorges Reservoir area and Yangtze riparian system include decreases in ecosystem’s productivity [38], changes to plankton and microbial community structure [39], and changes to nutrient characteristics [40]–[43].

In this study, we aim to identify the key climatic drivers of JEV transmission in Chongqing from 1997 to 2008. We will also explore the spatial and temporal patterns of JE incidence, as well as the trends of climatic variables in Chongqing.

## Methods

### 2.1. Study sites and data collection

**Figure 1:**
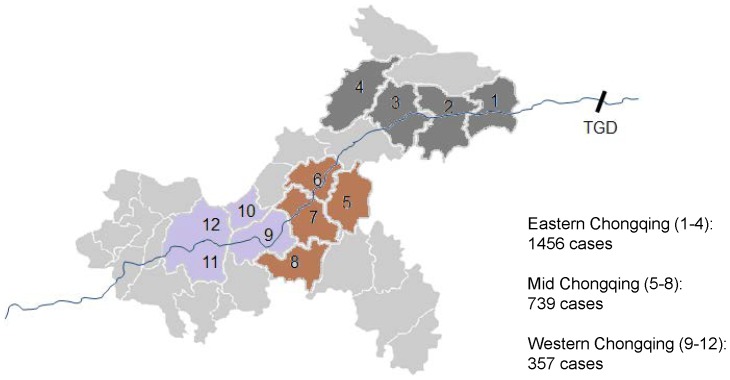
Spatial distribution of JE in the three geographic regions from 1997 to 2008. 1. Wushan (111.0 km); 2. Fengjie (149.0 km); 3. Yunyang (221.1 km); 4. Kaixian (252.6 km); 5. Shizhu (292.2 km); 6. Zhongxian (291.0 km); 7. Fengdu (332.8 km); 8. Wulong (355.2 km); 9. Fuling (370.3 km); 10. Changshou (392.7 km); 11. Yubei (439.0 km); 12. Banan (459.6 km). The numbers in brackets indicate the distance of the disctricts to the dam. TGD: the Three Gorges Dam.

This study was carried out in the following districts, all of which are located along the Yangtze River (Figure 1): Banan, Changshou, Fengdu, Fengjie, Fuling, Kaixian, Shizhu, Wulong, Wushan, Yubei, Yunyang and Zhongxian. The study area is located between 29°02’– 31°41’ north, and 105°11’–110°11’ east. The study area covers 33,312 square kilometers and contains a population of approximately 10.78 million, which is smaller than the city of Chongqing, with 82,000 square kilometers and a population of 31.44 million. The Yangtze River runs through the city from west to east and covers a total distance of 665 kilometers from end to end. Chongqing is located in a subtropical monsoon climate zone and the weather is humid. In the study area, the relative humidity is around 75%. Annual precipitation is heavy, peaking during late spring and summer, and the annual average rainfall ranges from 1000 to 1400 millimeters. The annual average temperature is around 18°C and the monthly temperature ranges between 5.3 – 31.9°C with a peak between July and August of 27.8 – 31.9°C. The annual hours of sunshine range from 1100 to 1500 hours total.

JE is an infectious disease that is routinely surveyed by the Center of Disease Control and Prevention (CDC), in China. The monthly reported cases from 1997 to 2008 for each district were new cases and were obtained from the CDC. The diagnosis of Japanese encephalitis is based on a combination of epidemiological data, clinical symptoms and signs, and specialized laboratory tests of blood, spinal fluid or Cerebrospinal fluid (CSF). These tests typically detect antibodies that respond to the viral infection. However, all confirmed cases have relied on antibody tests or pathogen isolation instead, and for this reason we decided to use the same approach of utilizing antibody tests and pathogen isolation in this study [44]. The antibody tests included IgM (serum and CSF) and IgG (serum) testing, whereas the pathogen isolation involved JE virus isolation from the CSF, or brain tissue or serum.

The CDC in China adopted several measures to ensure the quality of data obtained during JE surveillance. First, it fully designed the surveillance scheme and provided training and technical guidance to its local branches on surveillance, data collection and data analysis. Second, the Chinese CDC directly supervised the quality of reported JE cases. If abnormal values were obtained, they were confirmed directly by the CDC – first by the local branch, and if necessary, by the main government branch. Third, a review of reported JE cases was regularly conducted to guarantee the integrity of the data.

The Chinese CDC also provided the monthly meteorological data, including average temperature (T), relative humidity (RH), rainfall, and sunshine duration (SD). The meteorological variables were recorded by the *National Climate Center*. As participants of the National Basic Research Program of China (973 Program) (Grant No. 2012CB955500) lead by Professor Qiyong Liu, the Chinese CDC established a sharing platform(http://124.127.202.241/Share/index.jsp)in 2012 to provide a variety of data services for all of the study participants.

The values of missing JE cases were 5.55%. Most of these values had occurred during the winters of 2004, 2006 and 2007. To account for this, we used the “expand” procedure with the “step” option of SAS to compute the missing values. To evaluate the difference among the regions of Chongqing, and to get more convincing data, we combined the data from 12 districts into three geographic regions (Eastern, Middle and Western regions) by summing the count of JE and averaging the data of climatic variables (Figure 1).

### 2.2. Spatial and temporal analysis of JE incidence

To explore the spatial pattern of JEV transmission, the average annual JE incidence was separately calculated for each district, each of the three geographic regions, and the total area from 1997 to 2008. The monthly JE incidence was calculated for each district and each geographic region of Chongqing city, and the epidemic curves for three geographic regions were plotted to explore the temporal pattern of JEV transmission from 1997 to 2008. The monthly cases of JE occurred in the three regions were plotted to explore the seasonal trend of JE occurrence. In this study, the incidence rate was the number of new JE cases per population in a given time period. The annual population data was also obtained from the sharing platform created by the Chinese CDC. The incidences for each district, each of the three geographic regions, and the total area were calculated using the population of the corresponding area at certain time period. The denominator was given as 100,000 persons per year or per month.

### 2.3. Statistical model

A simple linear regression model was conducted to examine the trend of climatic variables over time from 1997 to 2008 in all three regions. To remove the seasonal effect, the regression was run using one month per year over the 10-year period and it was done for all 12 months separately.

For the main analysis, the response variable is the monthly JE incidence rate. Since it had a certain frequency of zero, a Zero-Inflated Poisson Regression (ZIPR) model was conducted to examine the association between the monthly JE incidence and the multiple climatic variables. In ZIPR, the count of disease is assumed to follow a mixture distribution of point-mass zero and a Poisson distribution, and the probability of the component and the mean parameter of Poisson distribution component are regressed on the covariates. Autocorrelation plots showed that the current incidence was significantly correlated with the incidence with a lag of 1, 11 and 12 months, and regressing the JE incidence on merely climatic variables resulted in highly auto-correlated residual terms. Therefore, we kept the JE incidence with a lag of 1, 11 and 12 months as covariates when studying the association between the incidence and the climatic variables.

We hypothesized that JE incidence was partially confounded by the improvement in the economic condition in region. So the annual gross domestic product (GDP) values of Chongqing city were included in the models to control for economic changes over time. We first ran the preliminary analysis to examine whether the GDP impacted the JE incidence, adjusting for the autocorrelation of the series. To do this, we ran the zero-inflated Poisson regression with incidence with a lag of 1, 11 and 12 months together with GDP values as covariates for the area as a whole as well as each of the three regions. Let y_t_ denote the count of JE at time t. The ZIPR assumed that y_t_  = 0 with probability p_t_, and y_t_ followed a Poisson distribution with mean µ_t_ with probability 1-p_t_. Instead of modeling the mean µ_t_, we modeled the incidence rate at time t rate_t_  = µ_t_/N_t_, where N_t_ denoted the population of a district at time t. Let rate_t-1_, rate_t-11_ and rate_t-12_ respectively denote the incidence rate with lag 1, 11 and 12 months, gdp_­t_ denote the GDP value. The preliminary analysis models for three regions were fitted as below:
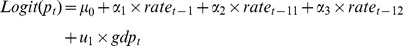


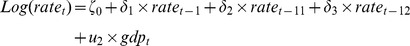



We then did the formal analysis to examine the relationship between JE incidence and climatic variables, adjusting for the autocorrelation of series and GDP. To do this, we checked the possible multi-collinearity among the different climatic variables with the different lags by examining the correlation matrix. It turned out that the temperature and sunshine series are highly correlated, so we just included temperature series as our predictor. In the final models, we included temperature and rainfall data due to their significant impact on the response.

Then we constructed the final model with following covariates: JE = incidence (denoted as “rate”) with lag = 1, 11 and 12; GDP series; Temperature (denoted as “temp”) and rainfall (denoted as “rain”) with possible lags. To determine lags, we used a forward model selection method, keeping the incidence with a lag of 1, 11 and 12 months together with the GDP values in the model and sequentially adding the lags of temperature or rain in the model; when each new term was added, we checked VIF to avoid the multicollinearity, and examined to make sure that the coefficients of climatic variables in the model were significant under level 0.05. We repeated this process until the log-likelihood of the fitted model, which provided a summary of model fitting, became stabilized. Once we determined the model for the whole area, we applied the model to three regions respectively. From the process of model selection, in addition to the GDP term and the incidence with lag 1, 11 and 12 months, we decided to include temperature of lag 1 and 3 months together with rain of lag 0 and 4 months in the model. Let rate_t-1_, rate_t-11_ and rate_t-12_ respectively denote the incidence with lag 1, 11 and 12 months, gdp_­t_ denote the GDP series, temp_t-1_, temp_t-3_ denote the temperature series with lag 1 and 3 months, rain_t_, rain_t-4_ denote rain series with lag 0 and 4 months. The models for the whole area are shown as following:
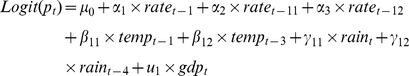


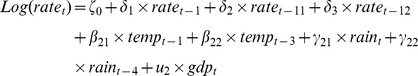



Autocorrelation plot and normal probability plot of the residuals were used to examine goodness-of-fit of the ZIPR models after each model was fixed. The Pearson residuals were expected to be uncorrelated and approximately normally distributed.

## Results

### 3.1. Spatial and temporal trend of JE incidence

A total of 2,552 cases of JE were reported from 1997 to 2008 in all 12 districts of Chongqing that we studied. The cumulative number of JE cases in each district from 1997 to 2008 ranged from 23 to 510 with a median of 151. The number of cases that occurred in eastern, middle and western parts of Chongqing city was 1456, 739 and 357 respectively. The spatial trend of JE cases decreased gradually from east to west (Figure 1).

**Figure 2:**
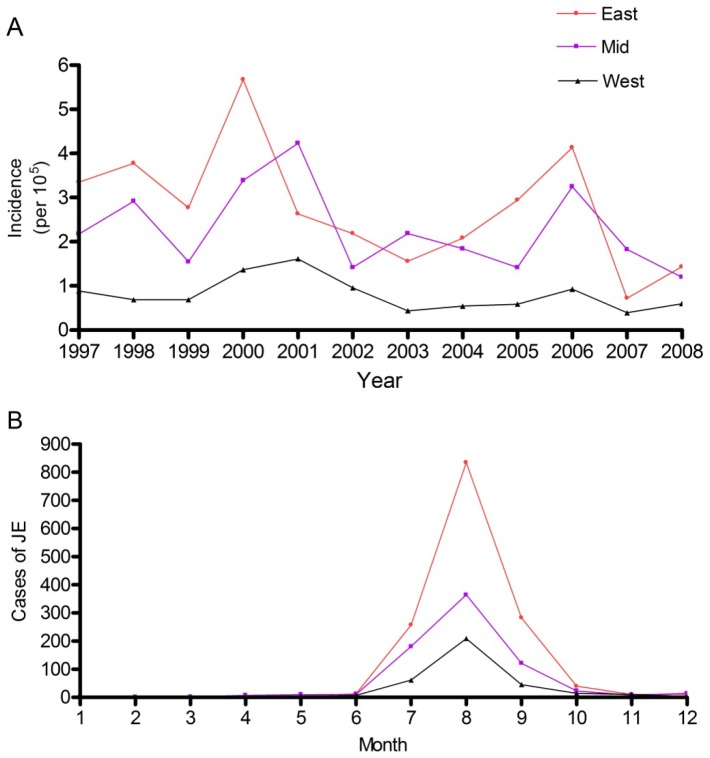
Temporal trend of JE occurrence from 1997 to 2008. (A) Annual JE incidence in the three geographic regions; (B) Monthly JE cases occurred in the three regions. East: Eastern Chongqing; Mid: Middle Chongqing; West: Western Chongqing.

In the study area, the annual JE incidence varied from 2.2/100,000 in 1997 to 0.7/100,000 in 2008 with a median of 1.8/100,000. The annual incidence peaked in 2000, reaching 3.6/100,000. The spatial variation in JE incidences over the three geographic regions showed that the annual incidence ranged from 0.4/100,000 (western in 2007) to 5.7/100,000 (eastern in 2000) with a median of 1.6/100,000 from 1997 to 2008. The JE incidence peaked in 2000 in the eastern region (5.7/100,000) and in 2001 in the middle region (4.2/100,000). In 2000, the annual incidence rapidly increased from 2.8/100,000 to 5.7/100,000 in eastern region, and from 1.5/100,000 to 3.4/100,000 in middle region. In 2006, the annual incidence reached the second peak from 2.9/100,000 to 4.1/100,000 in the eastern region, and from 1.4/100,000 to 3.2/100,000 in the middle region. Nevertheless, the annual incidence remained at relatively low levels in the western region, with a mean of 0.8 ± 0.4/100,000 (mean ± standard deviation) from 1997 to 2008 (Figure 2A).

The occurrence of JE had a clear seasonal trend in all regions of Chongqing. In total, the number of JE cases in the first half year (January to June) remained at quite low levels and even bottomed out at zero for some time. Beginning in June, the reported number of JE cases rapidly increased, peaking in August before decreasing again in September. By October or November in some districts, the reported cases rapidly decreased back to the low levels (Figure 2B). The cases of JE reported in August were more than 53.44% of the total cases that occurred in the three geographic regions of Chongqing between 1997 and 2008, while the cases of JE reported between July and September were more than 91.41% of the total of cases that occurred in the three geographic regions of Chongqing from 1997 to 2008.

### 3.2. The seasonal fluctuation of climatic variables

**Figure 3:**
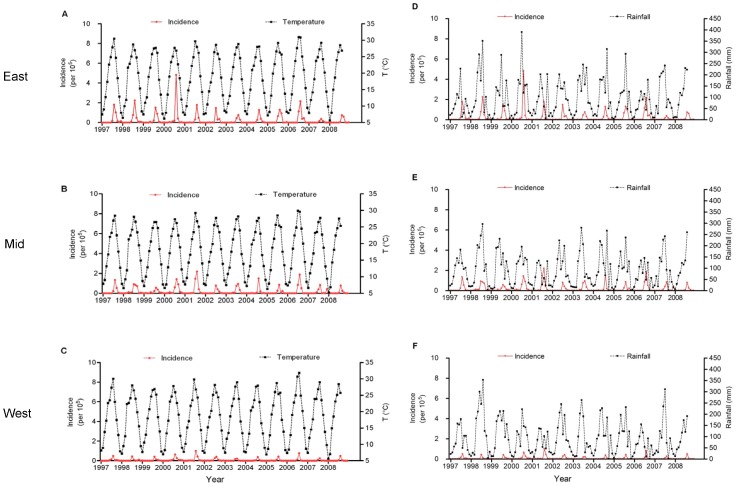
Monthly JE incidence and climatic variables in different regions from 1997 to 2008. (A) Monthly JE incidence and average temperature in eastern Chongqing; (B) Monthly JE incidence and average temperature in middle Chongqing; (C) Monthly JE incidence and average temperature in western Chongqing; (D) Monthly JE incidence and average rainfall in eastern Chongqing; (E) Monthly JE incidence and average rainfall in middle Chongqing; (F) Monthly JE incidence and average rainfall in western Chongqing. East: Eastern Chongqing; Mid: Middle Chongqing; West: Western Chongqing.

In the three regions, the monthly temperature ranged approximately from 5 to 8.5°C in January, then increased gradually by up to 27.7–31.9°C in July or August and decreased gradually from September to the next January (Figure 3A-C). The annual precipitation in Chongqing ranged from 1000 to 1400 mm and the heavy period of rainfall began in May and extended through October in the study area (Figure 3D-F). A simple linear regression model revealed that the temperature and SD in March and April tended to increase, and the temperature and SD in September and October tended to decrease through the studied period in all three regions (Table 1–2); the rainfall in September also tended to decrease in all three regions, and the rainfall in middle region also decrease in October compared with October and November in western region. Only in the western region, specifically between March and June, did rainfall tend to increase (Table 1–3). There was no significant monthly trend of RH in the middle and eastern regions from 1997 to 2008 (Table 1–2). However, the RH in March, April and May decreased significantly from 1997 to 2008 in the western region of Chongqing (Table 3).

**Table 1:** Simple linear regression coefficients of the monthly climatic variables over the time (1997–2008) in eastern Chongqing.

Month	T	RH	Rainfall	SD
January	0.5757	–0.7736	0.0968	0.4941
February	1.7777	–0.9902	0.8595	1.9745
March	3.1631**	–0.4893	1.7103	3.3140**
April	2.9985**	–0.8886	1.5079	3.2037**
May	2.0231	–2.1080	0.6932	2.5354*
June	0.1524	1.9614	1.7483	0.2284
July	–0.6251	–0.4166	0.8195	–1.3966
August	–1.4153	–0.9220	–1.4364	–0.6257
September	–4.7573***	–1.7396	–3.0824**	–3.1782**
October	–3.7927***	0.5671	–1.6556	–3.9479***
November	–1.3569	1.1671	–1.3460	–1.6419
December	0.0303	1.6075	–1.0251	–0.1268

T: average temperature; RH: relative humidity; SD: sunshine duration. *p≤0.05, **p≤0.01, ***p≤0.001.

**Table 2:** Simple linear regression coefficients of the monthly climatic variables over the time (1997–2008) in the middle region of Chongqing.

Month	T	RH	Rainfall	SD
January	0.4552	–0.5588	0.1515	0.5371
February	1.6385	–1.0961	1.4192	2.0542
March	3.1030**	–0.9689	1.7543	3.4359**
April	2.7998*	–2.1578	1.4771	3.4948**
May	1.7876	–2.1705	0.1171	2.2140*
June	0.1580	1.3125	1.9869	0.5271
July	–0.6712	–0.7876	–0.0102	–1.1510
August	–1.5422	0.0941	–0.7275	–0.8591
September	–4.5770***	–0.0332	–2.9418*	–2.5712*
October	–3.5901**	–0.3751	–2.3973*	–2.7427*
November	–1.3460	–0.2907	–1.8985	–1.4752
December	–0.0341	–0.2723	–0.8396	–0.7386

T: average temperature; RH: relative humidity; SD: sunshine duration. *p≤0.05, **p≤0.01, ***p≤0.001.

**Table 3:** Simple linear regression coefficients of the monthly climatic variables over the time (1997–2008) in western Chongqing.

Month	T	RH	Rainfall	SD
January	0.4585	–0.4722	0.1430	0.4112
February	1.6264	–0.4861	1.3353	1.3497
March	3.1344**	–2.4113*	2.2005*	3.5753**
April	2.8649*	–2.5246*	1.0391	4.0579***
May	1.8619	–2.7487*	0.5696	2.7538*
June	0.2456	–0.0648	2.5497*	1.4173
July	–0.6162	–1.2621	–0.1114	–0.8580
August	–1.4706	–0.2045	–0.7345	–0.8212
September	–4.4359***	–0.0739	–3.7010***	–2.1121
October	–3.6409**	–0.2576	–2.6563*	–3.3652*
November	–1.2812	–0.0475	–2.4575*	–1.8904
December	–0.0778	–0.2924	–0.4476	–1.1672

T: average temperature; RH: relative humidity; SD: sunshine duration. *p≤0.05, **p≤0.01, ***p≤0.001.

### 3.3. The relationship of GDP and JE incidence

To assess the impact of GDP on JE incidence, we first ran a preliminary ZIPR analysis including GDP as the predictor. We observed a significant positive association between GDP and the zero point-mass component of incident rate in the middle district (p<0.05), as well as a marginally significant negative association between GDP and the incidence of the disease for the Poisson component in the east district (p>0.05) (Table 4). These results implied an overall negative association between GDP and JE incidence in these two districts. Therefore, we decided to include GDP in the final model.

**Table 4:** The association between GDP and JE incidence in different regions from 1997 to 2008. Coefficients of GDP in the Zero-inflation Poisson Regression Model (preliminary analysis).

Region	Poisson	Zero-inflated
All	0.0005**	0.0059**
Eastern	–0.0003**	0.0008
Middle	0.0003	0.0030**
Western	0.0004	0.0023

All: the whole study area; Eastern: Eastern Chongqing; Middle: Middle Chongqing; Western: Western Chongqing. *p≤0.1, **p≤0.05.

### 3.4. Association between climate, GDP and JE incidence

A final ZIPR model was run to determine the association between climatic variables and JE incidence across different geographical regions, controlling for GDP (results shown in Table 5). The data showed positive coefficients of temperature with a lag of 1 and 3 months and positive coefficients of temperature with a lag of 1 month in the model, which indicated a positive association between the temperature with a lag of 1, 3 months and the incidence of the disease for the Poisson component as well as the positive association between temperature with a lag of 1 month and the probability of the point-mass component of zero in the incidence. All in all, there was an overall positive association between the temperature with a lag of 3 months and JE incidence. A higher value of temperature with a lag of 1 month was associated with a higher probability of observing a zero and with a higher mean incidence for the Poisson component of the mixture, and we couldn’t determine whether the overall association between temperature with a lag of 1 month and incidence was positive or negative. Also, we obtained negative coefficients of rain with a lag of 0 and 4 months in model. This implied a negative association between the rain with a lag of 0 and 4 months and JE incidence for the Poisson component of mixture. Therefore, there was an overall negative association between the rain with a lag of 0 and 4 months and the incidence of disease. Also, we observed the following patterns by region:

**Table 5:** The association between climatic variables and JE incidence controlling for GDP by region from 1997 to 2008, based on ZIPR models.

	Poisson Component			Zero-Point-Mass Component		
Region	T	Rainfall	GDP	T	Rainfall	GDP
All	L1 = 0.2984***	L0 = –0.0008**	L0 = –0.0016***	L1 = 0.3346*	L0 = –0.0035	L0 = 0.0074*
	L3 = 0.0364***	L4 = –0.0039***		L3 = 0.2032	L4 = –0.0120	
Eastern	L1 = 0.3754***	L0 = –0.0018***	L0 = –0.0028***	L1 = 0.2713	L0 = –0.0044	L0 = –0.0008
	L3 = 0.0766** *	L4 = –0.0077***		L3 = 0.0984	L4 = –0.0122	
Middle	L1 = 0.2687***	L0 = –0.0031***	L0 = –0.0006*	L1 = –0.0085	L0 = –0.0185	L0 = 0.0078*
	L3 = –0.0225	L4 = –0.0045***		L3 = –0.0398	L4 = –0.0611*	
Western	L1 = 0.2369***	L0 = 0.0006	L0 = –0.0013**	L1 = 0.1363	L0 = 0.0033	L0 = 0.0006
	L3 = 0.1066***	L4 = –0.0064***		L3 = 0.2911*	L4 = –0.0228*	

All: the whole study area; Eastern: Eastern Chongqing; Middle: Middle Chongqing; Western: Western Chongqing. Lx: the lagged months. T: average temperature, *p≤0.05, **p≤0.01, ***p≤0.001.

Eastern region: There was an overall positive association between the temperature with a lag of 1 and 3 months and JE incidence, and an overall negative association between the rain with a lag of 0 and 4 months and JE incidence.

Middle region: There was an overall positive association between the temperature with a lag of 1 month and JE incidence, and an overall negative association between the rain with a lag of 0 month and JE incidence.

Western region: There was an overall positive association between the temperature with a lag of 1 month and JE incidence.

The diagnostic plots in the fitted models demonstrated little evidence of serious deviation from the model assumption (Figure S1).

## Discussion

In this paper we conducted a ZIPR model to identify key climatic variables that had a close association with JE incidence in Chongqing between 1997 and 2008. By comparing the incidence of JE among different districts we described the spatial and temporal trend of JEV transmission in Chongqing. We also identified the fluctuation of major meteorological factors during this period.

It has been suggested that the temperature and rainfall were crucial to JE virus incubation and transmission [17], [30]. Therefore, we used a ZIPR model to analyze the association between these two factors and the incidence of JE. The data showed that there was a positive association of temperature with a lag of 3 months with JE incidence in Chongqing, which further verified the findings of previous investigations. *Culex. tritaeniorhynchus* is the main vector of the JE virus in the study area, and its life cycle varies depending on the temperature. Temperature can influence JE incidence through affecting the development of larvae, thereby helping to facilitate or impede the spread of the virus. Our findings on temperature (with a lag of 3 months for the JE incidence) indicate that it takes 3 months for temperature to influence the occurrence of JE in Chongqing.

However, our discovery that the rainfall with a lag of 0 and 4 months has a negative association with JE incidence is contrary to other publications that showed positive association between rainfall and JE incidence [30], [34], [45]. To an extent, precipitation would be crucial for the propagation and development of the mosquitoes, which would help the virus spread. But, heavy rainfall may also hold the potential to destroy existing mosquito breeding sites or interrupt the development of mosquito larvae [46]. Chongqing is located in a subtropical monsoon climate zone and annual total precipitation is heavy, ranging from 1000 to 1400 millimeters. This could well explain why in our study the rainfall was inversely related to JE incidence. Some studies have been shown to support the inverse relationship between rainfall and larval mosquito abundance [31], [32]. In our case, the heavy rainfall rapidly began in May and lasted to September or October. Considering that the rainfall with a lag of 4 months had a negative association with JE incidence, these results could explain why JE incidence declined beginning in September or October.

In our study, the number of JE cases that occurred between July and September accounts for 91.41% of the total number; the number of JE emerging only in August accounts for 53.44% of the total number from 1997 to 2008. This seasonal trend could be explained by interplay of temperature with a lag of 1 month with rainfall with a lag of 4 months, which may affect the density of mosquitoes and pigs breeding as well as other vectors required for the cycle of the JE virus.

Comparing with the whole study area, there were similar results in the association between temperature and rainfall and JE incidence in the three regions of Chongqing. Except for temperature with a lag of 3 months, in both the eastern and middle regions temperature with a lag of 1 month was also positively related to JE incidence. This finding indicated that temperature affected the JE incidence through not only a long-term control mechanism but also a short-term direct mechanism. However, there seemed to be no significant association between the temperature of the current month and the JE incidence in this study.

JE cases appeared mostly in the eastern and middle regions of Chongqing from 1997 to 2008, probably due to different economic status among these regions. Interestingly, the incidence of JE gradually increased with increased proximity to the TGD. Even though some negative effects of TGD on ecosystem have been emphasized [38], [47], the direct impact of TGD on infectious diseases is still not well verified [48]–[50]. In our study, we monitored the fluctuation of climatic variables during the period of the TGD construction from 1997 to 2008. We revealed that the temperature during March and April tended to increase, while the temperature during September and October tended to decrease – as did the rainfall during September in three regions of Chongqing. However, the effects of dam construction on the changes of climatic variables and in turn on the JE incidence must be further studied. At least by now, the negative impact of TGD on JE incidence might be too slight to neutralize the positive effects of improved sanitation and economic development in Chongqing.

Although the JE incidence tended to decrease in both Chongqing and individual regions, there were still two evident peaks that emerged in 2000, 2001, and 2006 in both the eastern and middle regions of Chongqing. The emergence of JE involved a complicated interplay of hosts, vectors, climatic variables and anthropogenic factors [16], [17]. In our study, the JE incidence peaked in the years when there was a relatively higher temperature and less precipitation. Further investigation in this area is required to confirm the role of temperature and rainfall in JEV transmission through ruling out any effects of other confounding factors such as movement of infected individuals into or out the regions.

Our study had limitations involving the collecting of data, spatial analysis of JE incidence, the fitting of the model to individual regions, and weak evidence linking the TGD and JE incidence.

In this study we didn’t take into consideration other potential factors that may affect JE incidence. For example, JEV is transmitted to human by mosquitoes, and domestic pigs. Birds can also facilitate the spreading of JEV. The effect of these vector and hosts in the study area were not included into our model. Rice field proportion, which may have been changed by the construction of the TGD, is also directly associated with the incidence of JE because these fields provide breeding sites for mosquitoes, which we did not take into account. The relocation of infected residents may also lead to the reoccurrence of JE incidence. And last, the GDP data we obtained was only related to Chongqing and not to all regions. Nonetheless, we have no evidence of any systematic biases in our analysis that were likely to lead us to the wrong conclusion.

In addition, it is important to point out that we separated the study area into three regions in order to observe spatial trends in JE incidence. Theoretically, different results could be obtained by reorganizing these regions, although similar results would be expected.

We also only focused on data from 1997 to 2008-the time during which the TGD was being constructed. Pre- and post-construction data were not available, so we could not analyze the impact of TGD on climatic variables or JEV transmission. Future analysis could be performed by including more complete data over a longer time period.

In conclusion, temperature with a lag of 1 and 3 months and rainfall with a lag of 0 and 4 months were key climatic variables that were associated with JE incidence in Chongqing from 1997 to 2008. The spatial and temporal trend of JE incidence occurring in Chongqing was associated with temperature and rainfall with different lags. The reasons for seasonal fluctuations of climatic variables are not well understood and needed to be further investigated. Future study is required to validate the findings of this study and further explore the effect of TGD on JE incidence.

## Supporting Information

Figure S1 **The diagnostic plots in the fitted model.** The plots shown here are for the fitted model of the study area. Those for the three regions are similar (not shown).
(TIFF)
