# Supplementary figures and images for: Regional Impact of Climate on Japanese Encephalitis in Areas Located near the Three Gorges Dam
Source: PLoS One. 2014 Jan 3;9(1):e84326. doi: 10.1371/journal.pone.0084326 (PMC3880291; doi:10.1371/journal.pone.0084326)

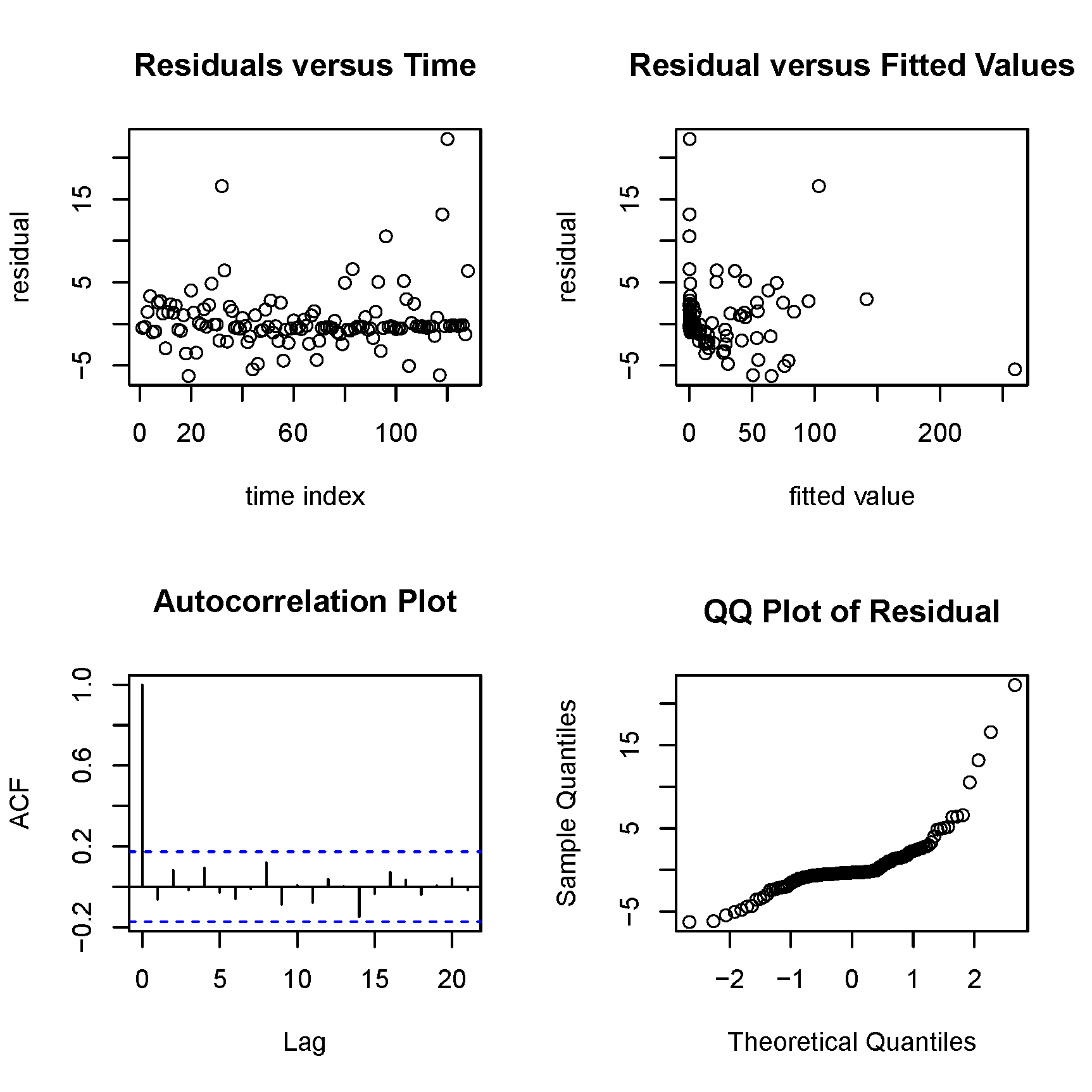

Supplement: Figure S1 — The diagnostic plots in the fitted model. The plots shown here are for the fitted model of the study area. Those for the three regions are similar (not shown). (TIFF) [file pone.0084326.s001.tiff]
